# Endogenous rhythms influence musicians’ and non-musicians’ interpersonal synchrony

**DOI:** 10.1038/s41598-022-16686-2

**Published:** 2022-07-28

**Authors:** Pauline Tranchant, Eléonore Scholler, Caroline Palmer

**Affiliations:** Department of Psychology, McGill University, 1205 Dr Penfield Ave, Montreal, QC H3A 1B1 Canada

**Keywords:** Computational biology and bioinformatics, Psychology

## Abstract

Individuals display considerable rate differences in the spontaneous production of rhythmic behaviors (such as speech, gait, dance). Temporal precision in rhythmic behavior tends to be highest at individuals’ spontaneous production rates; musically trained partners with similar spontaneous rates show increased synchrony in joint tasks, consistent with predictions based on intrinsic frequencies of coupled oscillators. We address whether partner-specific influences of intrinsic frequencies are evidenced in musically trained and untrained individuals who tapped a familiar melody at a spontaneous (uncued) rate individually. Each individual then synchronized with a partner from the same musicianship group at an initially cued rate that matched the partners’ spontaneous rates. Musically trained partners showed greater synchrony in joint tapping than musically untrained partners. Asynchrony increased in both groups as the partners’ difference in individual spontaneous rates increased, with greater impact for musically untrained pairs. Recurrence quantification analysis confirmed that musically untrained individuals demonstrated greater determinism (less flexibility) in their tapping than musically trained individuals. Furthermore, individuals with greater determinism in solo performances demonstrated reduced synchrony in joint performances. These findings suggest that musicians’ increased temporal flexibility is associated with decreased endogenous constraints on production rate and greater interpersonal synchrony in musical tasks.

## Introduction

Humans have a refined capacity to synchronize their actions with auditory rhythmic signals. Interpersonal synchrony is a special case of synchronization that refers to temporal coordination between individuals. Interpersonal synchrony is crucial for many social contexts such as dance, team sports, and conversational speech. Greater synchrony between individuals is associated with increased affiliation between partners^1,2^ and more cooperative behaviour^3–5^. Music performance is an especially precise form of interpersonal coordination. Performers must adapt their production of tone sequences based on auditory information from themselves and their partners to achieve synchrony^6,7^. Previous research showed that endogenous rhythms constrain this process: Greater synchrony between partners was predicted by smaller differences in their spontaneous production rates^8,9^. The relationship between interpersonal synchrony and spontaneous rates also extends to larger groups performing a rhythmic task: Greater synchronization in individuals’ hand movements was observed in seven-person groups among individuals whose spontaneous movement rates were similar^10^. Spontaneous rates of rhythmic actions in locomotion tasks (walking, running) in several species correspond to rates that require minimum energy expenditure, reflecting a state of optimal efficiency^11,12^. Consistent differences in human spontaneous rates are evidenced in a variety of rhythmic behaviors including speech^13,14^, hand clapping^15^, and finger tapping^16–18^. The relationship between interpersonal synchrony and spontaneous rates has been measured primarily in individuals with significant behavioral expertise in the measured tasks. We address whether musically untrained individuals show reduced synchrony in a novel musical task when they differ in their spontaneous production rates.

Constraints of spontaneous rates on interpersonal synchrony have a natural explanation in a nonlinear dynamical systems theory of internal timing and movement synchronization^19^. Spontaneous production rates are thought to reflect the intrinsic or natural frequency of an oscillator acting as an attractor toward which a system will converge over time^20,21^ and whose signature can be captured at a neural level^22^. Synchronization between interacting individuals has been modelled by the coupling of oscillators that adapt in frequency and relative phase^23–25^. Oscillators with similar intrinsic frequencies will be faster to adjust and more strongly coupled, resulting in higher synchronization accuracy. We test this hypothesis by comparing partners’ tapping synchronization in a joint musical task with their intrinsic frequencies as measured by their spontaneous rates in a solo task.

Musical training also impacts interpersonal synchrony. Individuals who received musical training show higher synchronization accuracy to external auditory rhythms than do untrained individuals^26–29^. Musicians also show lower temporal variability than non-musicians when they produce rhythmic sequences in the absence of an external cue^30,31^. Musically trained individuals adapt their synchronization performance more quickly in response to changing auditory rates^32,33^. Finally, musical training enhances individuals’ flexibility to produce sequences at rates other than the spontaneous rate^31,34^, indicating a reduced attractor strength of intrinsic frequencies during adaptation to external auditory signals. We test here the combined influence of spontaneous rates and musical training on partners’ asynchronies in a music tapping task that permits non-musicians to produce music without any required training.

Two different tasks have measured natural movement rates for rhythmic sequences in previous studies: Spontaneous Motor Tempo (SMT) and Spontaneous Production Rate (SPR). The SMT task consists of producing temporally regular tapping with one finger of the dominant hand in the absence of auditory feedback, whereas the SPR task consists of producing simple melodies (with one or more fingers) in the presence of auditory feedback. SMT has been used in numerous studies that investigate human timing, whereas SPR has been used with music-evoking movement. Age, physiological arousal, and time of day have been shown to influence SMT^16,35,36^, factors that show reduced influence on SPR^31,37^. A novel musical task was recently developed to measure SPR in both musicians and non-musicians^31^, making possible the measurement of SPR in untrained individuals. That task showed that non-musicians were more rigid and less flexible in their synchronization of music-generating finger taps with a metronome, as evidenced by recurring timing patterns measured with Recurrence Quantification Analysis (RQA). We extend this analysis to test interpersonal synchrony between partners (musician pairs and non-musician pairs) who synchronize with their partner; specifically, we test the hypothesis that RQA techniques should indicate greater rigidity in non-musicians’ interpersonal timing.

Pairs of musically trained participants (n = 14 pairs) and pairs of untrained participants (n = 14 pairs) participated in the experiment. Spontaneous rate measures of participants’ SMT and SPR were first collected individually (Solo tasks), and then Duet measures of synchrony were collected from each pair while they tapped melodies together at an initially cued rate that corresponded to the spontaneous rates (SPR) of each partner. Their taps were recorded by a force sensor that allowed listeners with or without musical training to produce melodies by simply tapping on the sensor (with one finger) to hear the next pitch in the melody^31^. We hypothesized that SPR and SMT reflect the frequencies of different intrinsic oscillations, with SMT measuring a motor (finger) frequency and SPR measuring an auditory-motor coupling frequency. Based on previous research with musicians^38^, we expected that the SPR difference between non-musician partners would predict synchronization accuracy and leader/follower patterns: Lower accuracy (larger magnitude of asynchronies) should result for both musician and nonmusician pairs whose Solo SPRs differ greatly, with the faster-SPR participant in each pair tapping ahead of their partner. Finally, we predicted that musically untrained pairs should exhibit larger asynchronies, larger temporal variability, and increased determinism (rigidity) in timing their joint synchronization, consistent with lower temporal flexibility, compared with musically trained pairs.

## Results

### Solo tasks: SPR and SMT measures are unrelated

**Figure 1:**
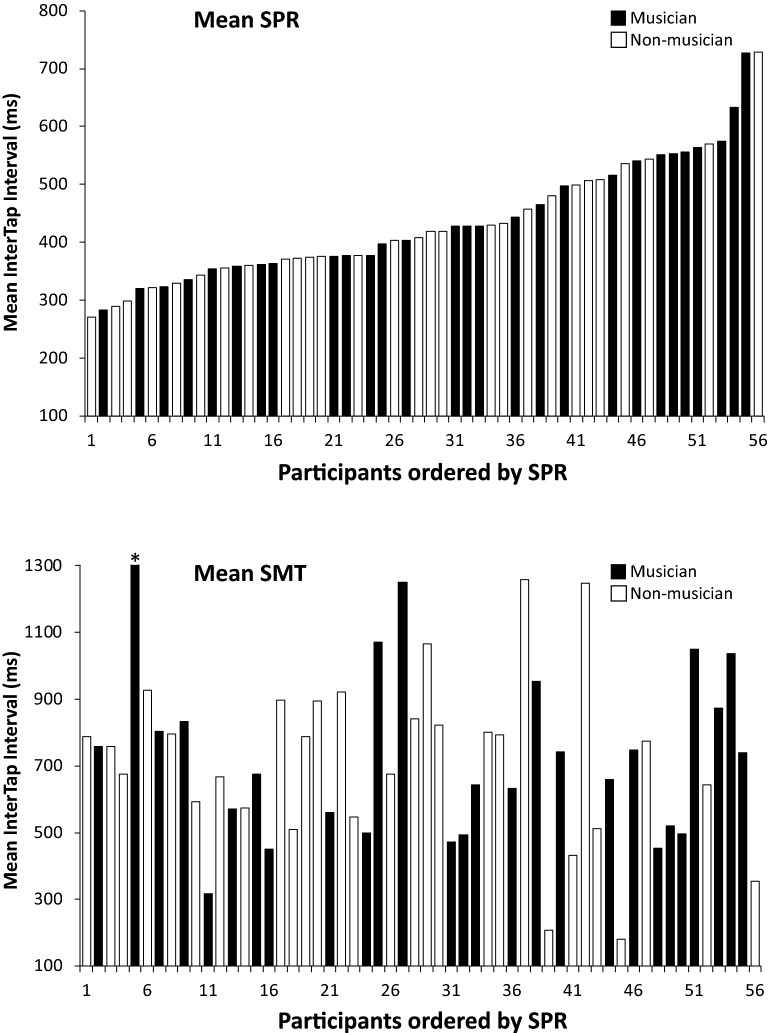
Mean Intertap interval in Solo production rate (SPR) task (top) and SMT task (bottom). Participants ordered left to right by increasing SPR value (top and bottom). Each bar represents one participant. Asterisk indicates an SMT value more than 3 standard deviations above the grand mean (= 2102 ms).

Figure 1 shows each participant’s mean tapping rates in the SPR and SMT tasks, ordered in both plots from fastest to slowest participant in the SPR task. A wide range of SPR values were obtained for musician and non-musician groups; the SMT values also showed wide inter-individual variation. There was no correlation between the SPR and SMT values across participants (*r* (54) = − 0.1625, *p* = 0.23) or within groups (Musicians: *r* (26) = − 0.09, *p* = 0.63; Non-musicians: *r* (26) = − 0.29, *p* = 0.13). Furthermore, SMT and SPR values did not differ by musical training or interact with task; overall, participants were faster in the SPR task (*M* = 434, SE = 13.8) than in the SMT task (*M* = 738, SE = 40), *F*(1, 54) = 44.99, *p* < 0.001, $\upeta _{{\text{p}}}^{{2}}$  = 0.45.

The coefficient of variation which measures temporal variability was compared across SPR and SMT tasks. The CV was correlated across the SMT and SPR tasks for all participants (*r* (54) = 0.50, *p* < 0.0001) as well as within groups (non-musician group, *r* (26) = 0.40, *p* = 0.04; musicians, *r* (26) = 0.36, *p* = 0.06). The musician group showed smaller CVs (*M* = 0.045, SE = 0.0017) than the non-musician group (*M* = 0.064, SE = 0.0033), *F* (1, 54) = 18.57, *p* < 0.001, $\upeta _{{\text{p}}}^{{2}}$ = 0.26). Thus, temporal variability but not mean rate was correlated across SPR and SMT tasks.

### Duet tasks: duet asynchronies are influenced by musical training and partners’ spontaneous rates

**Figure 2:**
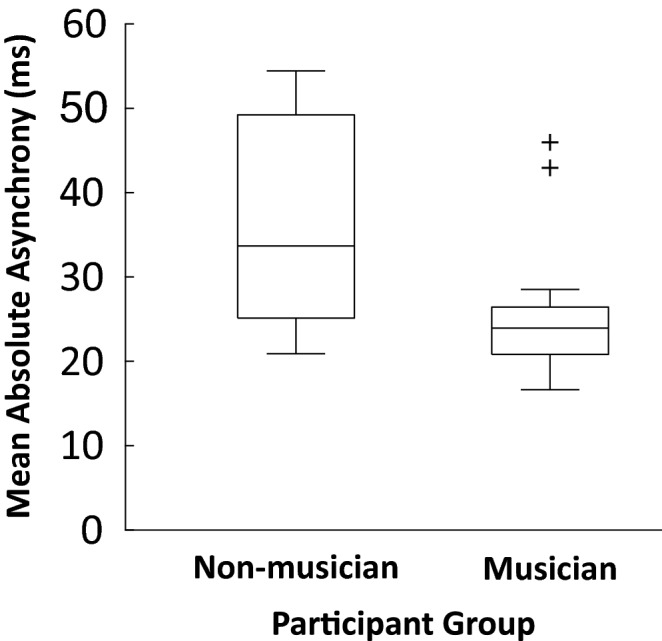
Mean absolute asynchrony values (ms) by participant group in the Duet performance task. Top and bottom whiskers indicate minimum and maximum values, respectively.

The partners’ mean absolute asynchronies in the Duet synchronization task were compared across groups. As shown in Fig. 2, musicians synchronized better with their partners (median = 23.94) than did non-musicians (median = 33.68, *U* = 150, *p* < 0.01). Group differences in temporal variability were also observed in the Duet condition; the CV was smaller for musicians (*M* = 0.049, *SE* = 0.0022) than for non-musicians (*M* = 0.064, *SE* = 0.0026, *F*(1, 54) = 19.66, *p* < 0.0001, $\upeta _{{\text{p}}}^{{2}}$ = 0.27). Finally, each participant’s CV in Solo and Duet conditions were correlated across all participants (*r* (54) = 0.6345*, p* < 0.001) as well as within groups (Non-musicians: *r* (26) = 0.55, *p* < 0.01; Musicians, *r*(26) = 0.63, *p* < 0.001), suggesting that temporal variability remained stable across Solo and Duet tasks.

**Figure 3:**
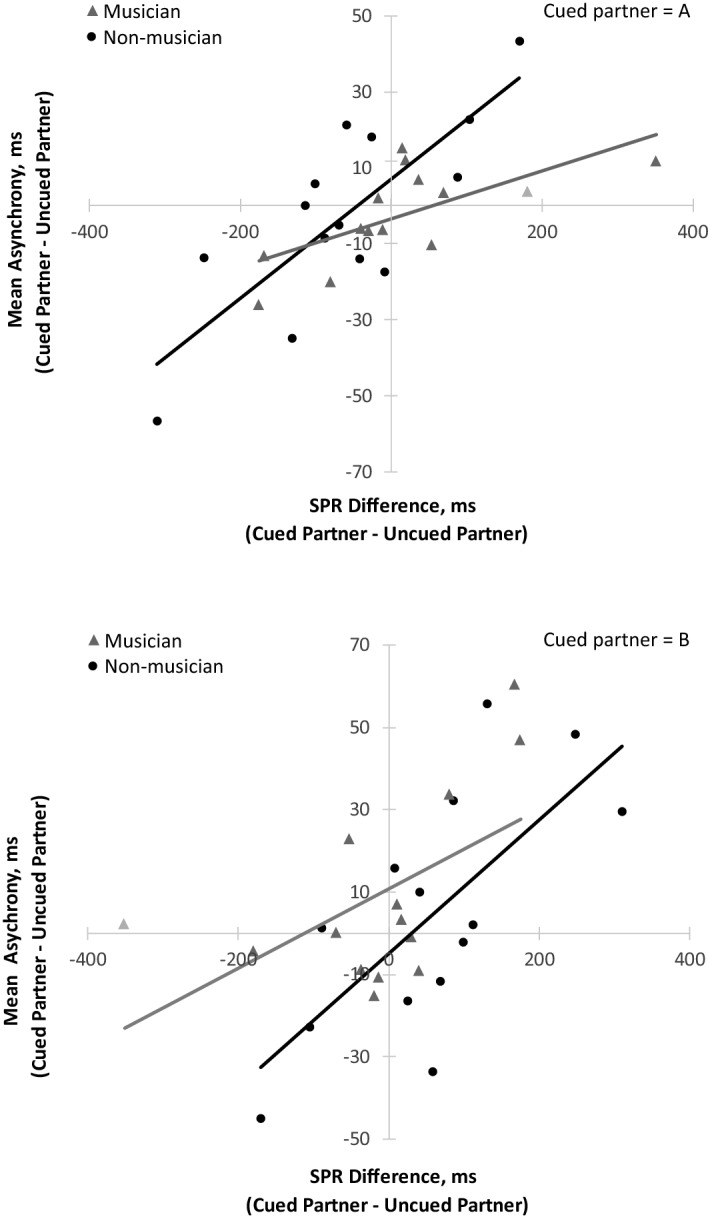
Duet synchronization task: mean asynchrony (cued partner–uncued partner, ms) plotted by partners’ Solo SPR difference (Cued partner–uncued partner, ms). Each marker represents one pair. Top: Condition A (cued rate = PartnerA), Bottom: Condition B (cued rate = PartnerB).

We test the dynamical systems prediction that each Duet pair’s signed asynchronies arose from the partners’ SPR differences. Figure 3 shows the mean signed asynchronies (cued partner’s onsets–uncued partners’ onsets) correlated with the partner’s Solo SPR Difference (cued partner’s rate minus uncued partner’s rate), separately for Condition A (metronome = PartnerA’s Solo rate) and Condition B (metronome = PartnerB’s Solo rate). Significant positive correlations were observed for all groups and all conditions: Condition A (*r* (12) = 0.79, *p* < 0.001 in non-musicians; *r*(12) = 0.68, *p* < 0.01 in musicians; Condition B (*r*(12) = 0.70, *p* < 0.01 in non-musicians; *r*(12) = 0.56, *p* < 0.05 in musicians). The larger the difference between partners’ SPRs, the larger their signed asynchronies; the partner with the faster Solo SPR generally anticipated the partner with the slower Solo SPR.

The regression slopes in Fig. 3 are larger (steeper) for non-musicians than for musicians, suggesting stronger constraints of endogenous rates on non-musician partners’ synchronization. To test the slope differences, a multiple regression analysis predicted the partners’ Duet asynchronies from the SPR differences, group membership (Non-musician coded 0; Musician coded 1), and the interaction of SPR differences with group membership. That regression was significant for both Condition A (*R* (24) = 0.77, *p* = 0.0001) and Condition B (*R* (24) = 0.65, *p* = 0.0035). The coefficient associated with SPR differences was again significant (Condition A: unstandardized coefficient = 0.1568, *p* = 0.00009; Condition B: unstandardized coefficient = 0.1620, *p* = 0.0016). Most important, the interaction term was significant for Condition A (unstandardized coefficient = − 0.0937, *p* = 0.0267) and showed a similar pattern in Condition B that did not reach significance (unstandardized coefficient = − 0.0655*, p* = 0.3105), indicating that the Non-musician slope was steeper than the Musician slope toward the beginning of the study session, consistent with the non-musicians’ less flexible adjustment to the cued metronome rate. Importantly, partners’ SMT differences did not predict duet asynchronies; no correlations between SMT differences and mean duet asynchronies reached significance (all *p’s* > 0.3).

### Recurrence quantification differs by musical training in solo performances

**Figure 4:**
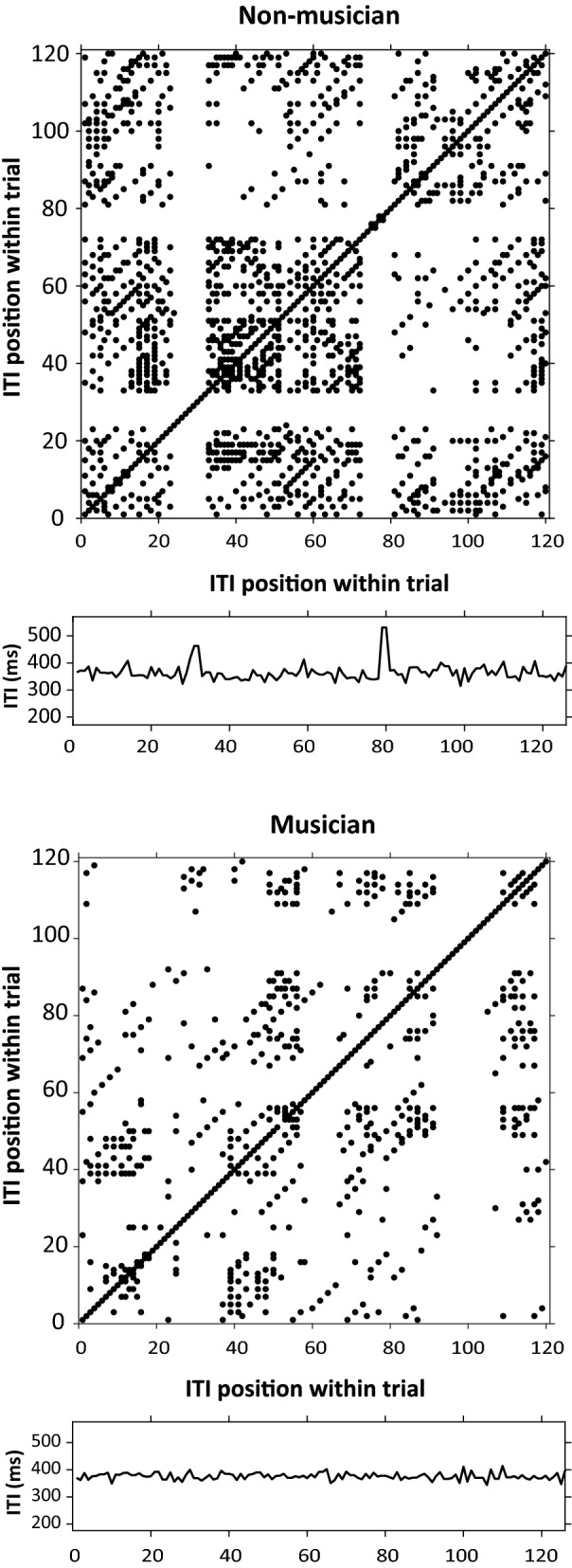
Recurrence plots demonstrating recurrence rate for intertap intervals (ITI) from sample trials of Solo spontaneous production rates. X- and y-axes = ITI time series from sample trial. Top: Non-musician: recurrence rate (represented by proportion of dots) = .0948. Bottom: Musician: recurrence rate = .0363.

Figure 4 shows recurrence plots for the intertap intervals from one Solo performance trial by a non-musician (top) and musician (bottom). The presence of black dots indicates a higher recurrence rate which was found for non-musicians (*M* = 0.1044, *SD* = 0.0958) than for musicians (*M* = 0.0529, *SD* = 0.0482), *U* = 562.5, *p* < 0.01). Thus, non-musicians exhibited higher patterning in the timing of their Solo tapping.

**Figure 5:**
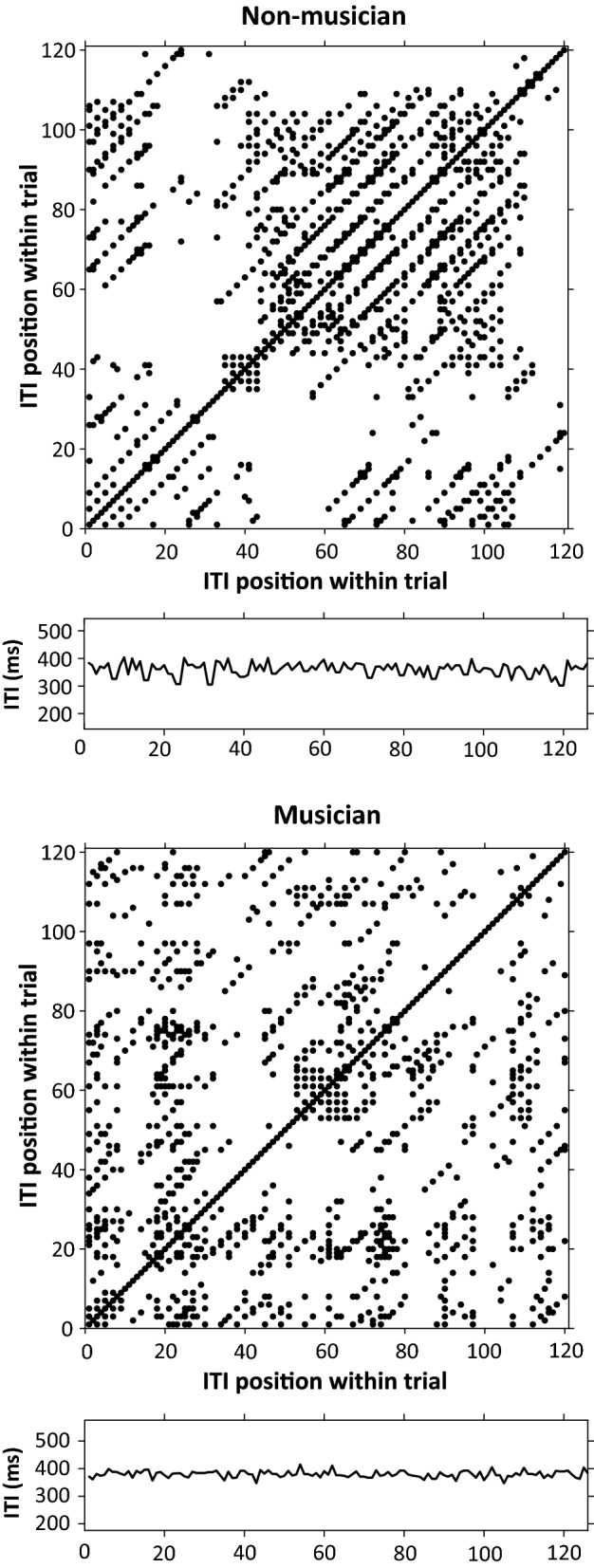
Recurrence plots demonstrating determinism for sample trials (intertap intervals) of Solo spontaneous production rates. X- and y-axes indicate ITI time series. Top: Non-musician: determinism (represented by % of points falling on diagonal lines) = .1842. Bottom: Musician: determinism = .0922.

Figure 5 shows plots of sample Solo performance trials that demonstrate high determinism (non-musician; top graph) and low determinism (musician; bottom graph), indicated by the presence of black dots forming diagonal lines that denote similar behavior over consecutive ITIs. The non-musicians’ ITIs exhibited higher determinism (*M* = 0.158, *SD* = 0.038) than did musicians (*M* = 0.1316, *SD* = 0.0268), *U* = 581, *p* < 0.001. Figure 5 (top) also shows the influence of the musical structure on the non-musician’s timing profile, captured by the distance between the diagonal lines. The participant’s diagonal lines were separated by distances of exactly 8 beats or taps, corresponding to the length of a musical subphrase (two metrical bars) in the simple melody performed. Thus, the RQA analyses illustrate constraints imposed by the melodic structure on the dynamics of nonmusicians’ tapping behavior.

**Figure 6:**
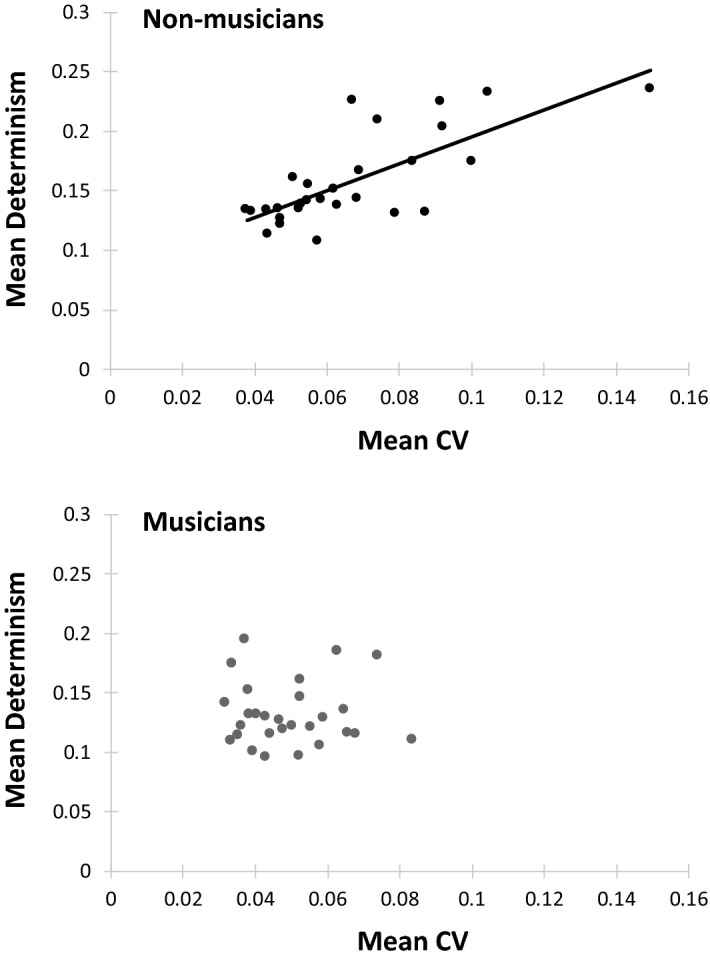
Solo performances: Mean CV (standard deviation / mean ITI) by mean determinism. Each dot represents one participant. Top: Non-musician group (r (26) = .74, p < .001). Bottom: Musician group (r (26) = − .003, p = .99).

Finally, we compared the temporal variability (CV) of the Solo performance ITIs with the determinism measures for the same individuals. As shown in Fig. 6, determinism increased as temporal variability increased for non-musicians (*r* (26) = 0.74, *p* < 0.0001) but not for musicians (*r* (26) = − 0.003, *p* = 0.99). Thus, the non-musicians’ overall increased variability corresponded to greater predictability or rigidity, rather than simply increased noise. There was no relationship between the CV and recurrence rate (unadjusted) across groups (*r* (54) = 0.1385, *p* = 0.31) or within groups (non-musicians: *r* (26) = 0.014, *p* = 0.94; musicians: *r* (26) = 0.079, *p* = 0.69).

### Recurrence quantification differs by musical training in duet performances

**Figure 7:**
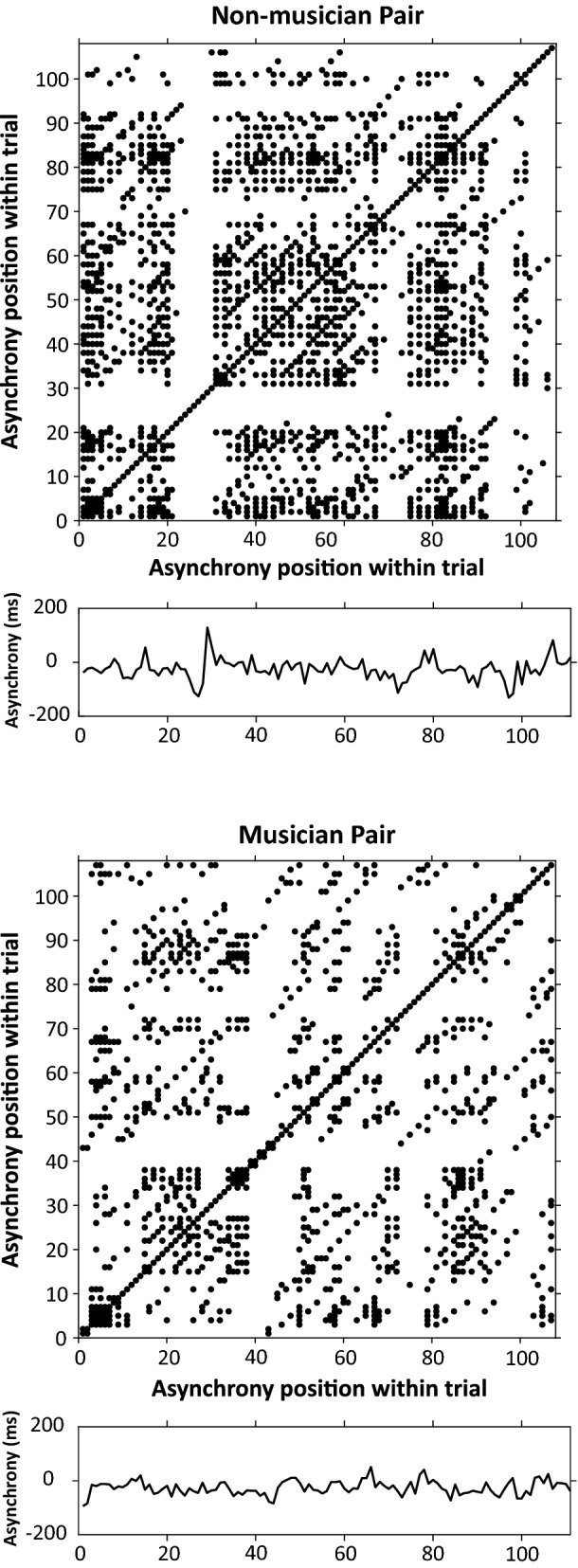
Recurrence plots demonstrating recurrence rate for sample trials (asynchronies) of Duet synchronization-continuation task. X- and y-axes indicate the asynchrony time series. Top: Non-musician pair’s recurrence rate (represented by percentage of points) = .1447. Bottom: Musician pair’s recurrence rate = .0878.

Next, we examine recurrence patterns in partners’ asynchronies in the Joint performances. RQA was computed on the signed asynchrony time series (asynchrony defined as Partner X tap onset − Partner Y tap onset, where the cued rate = Partner X’s SPR). Figure 7 shows a sample trial of asynchronies and the corresponding recurrence plot from a non-musician pair (top) and a musician pair (bottom). The mean recurrence rate was higher overall for non-musicians (*M* = 0.1236, *SD* = 0.0459; Median = 0.11) than for musicians (*M* = 0.0997, *SD* = 0.0268; Median = 0.09), *U* = 134, *p* = 0.049, indicating that the non-musicians’ asynchronies were more patterned than those of the musicians as they synchronized with a partner. Determinism values did not differ between the groups, *U* = 106, *p* = 0.14.

We compared the RQA measures of Duet asynchrony with behavioral variability by correlating the recurrence rates of each pair’s asynchronies with the standard deviation of that pair’s Duet asynchronies. The recurrence rates of the non-musicians’ asynchronies were significantly correlated with the SD of asynchronies for both conditions (PartnerA cue: *r* (12) = 0.6871, *p* = 0.0066; PartnerB cue: *r* (12) = 0.612, *p* = 0.02). The correlations were not significant for the musicians (PartnerA cue: *r* (12) = 0.2097, *p* = 0.4916; PartnerB cue, *r* (12) = 0.2101, *p* = 0.4906). Determinism values did not correlate with standard deviations of the joint asynchronies for either group. Similar to the Solo performance timing, non-musicians showed increased temporal variability in Duet asynchronies that coincided with increased (non-random) patterning of their asynchronies.

### Duet synchrony is predicted by partners’ solo performance recurrence patterns

Finally, the partners’ synchronization in the Duet performances was predicted from the recurrence patterns in each partner’s intertap intervals in Solo performances. Consistent with the directionality of the faster/slower partners’ Solo rate influences on the asynchronies (Fig. 3), each pair of partners was coded in terms of which partner had the faster/slower Solo rate. A multiple regression analysis predicting the partners’ Duet absolute asynchronies from each partner’s Solo recurrence indicated a significant overall fit, *R* (25) = 0.5020, *p* = 0.02. Partners with higher recurrence patterning (less flexibility) in their Solo timing patterns had greater difficulty synchronizing with their partner in Joint performances. The partner with the faster SPR tended to exhibit higher recurrence in their intertap intervals that contributed most to the pair’s asynchrony (faster partner: standardized coefficient = 0.4761, *p* = 0.01; slower partner: standardized coefficient = 0.1644, *p* = 0.35). The determinism values from the partners’ Solo performances (when recurrence rate was fixed) did not predict partners’ Duet asynchronies.

## Discussion

We examined intrinsic constraints that influence both musically trained and untrained individuals’ auditory synchrony as they produced musical sequences. Musicians and non-musicians performed an auditory-motor synchronization paradigm that permitted non-musicians to produce music without any required training. Interpersonal synchrony measures from partners who tapped a melody together showed significant constraints of the partners’ spontaneous production rates: Greater synchrony between partners was predicted by smaller differences in their individual spontaneous performance rates. The fact that both participant groups showed constraints of spontaneous rates on interpersonal synchrony supports the view that spontaneous rates are not learned but instead are intrinsic to rhythmic movement^32,39^. Spontaneous rates of rhythmic actions often correspond to movements that require minimum energy expenditure, reflecting a state of optimal efficiency^12,40,41^. Musically untrained adults showed greater constraints of these endogenous rhythms on interpersonal coordination than did trained adults, consistent with findings that flexibility increases as individuals achieve task expertise^31,34,42^. Nonlinear analyses of the intertap intervals (Solo) and asynchronies (Duet) confirmed greater recurrence (predictability) in non-musicians’ tapping, consistent with a multi-dimensional phase space in which movement trajectories change as individuals acquire auditory-motor expertise or additional sensorimotor information^43^.

Spontaneous (uncued) production rates have a natural explanation in nonlinear dynamical systems theory that an intrinsic frequency of an oscillator acts as an attractor toward which the system’s behavior will converge over time^20,21^. Synchronization between interacting individuals has been modelled with the coupling of oscillators that adapt in frequency and phase^23–25^. Oscillators with similar intrinsic frequencies are faster to adapt and more strongly coupled, resulting in higher synchronization accuracy, a theoretical prediction supported by analyses of partners’ tapping synchronization accuracy as a function of the difference in the partners’ spontaneous rates of solo productions. Visuomotor coordination tasks have shown that, as the difference in rate between an individual’s oscillatory movements and a rhythmically oscillating stimulus decreases, individuals are more likely to spontaneously entrain their movements to the stimulus rate^44,45^. Temporal variability measures in visuomotor coordination tasks have been linked to motor flexibility: Individuals who exhibited some temporal variability were more likely to unintentionally synchronize their movements with a wider range of stimulus rates^46^, consistent with findings in auditory-motor coordination tasks that the same individuals who show better synchronization tend to show enhanced flexibility across movement rates^31^.

Musical training modulated the strength with which the spontaneous rates influence interpersonal synchrony: Individuals with musical training showed higher synchronization accuracy to their partner’s intrinsic frequency than did untrained individuals^26–29^. Musicians also showed lower temporal variability than non-musicians when producing rhythmic sequences in absence of an external cue, also consistent with previous findings^30,31^. This is the first study to tie the non-musicians’ increased recurrence patterning to the amount of asynchrony, suggesting that temporal variability of non-musicians is not simply motor noise but instead reflects repetitive patterns that reduce one’s flexibility and adaptation when synchronizing with a partner. The non-musicians’ deterministic recurrence analysis also revealed the musical structure of the melody task, based on the spacing between the diagonal lines (Fig. 6). Overall, the non-musicians’ tapping was more strongly patterned by the musical structure of the time series.

Spontaneous Motor Tempo, a common measure of motor timing in the absence of auditory feedback, did not predict performance in the interpersonal synchronization task. SMT also did not correspond to measures of SPR, which did predict performance in the synchronization task. Despite large individual differences in both SMT and SPR measures (Fig. 1), there was no correlation across the tasks for musically trained or untrained individuals. Furthermore, individual SMT measures did not predict Duet performance synchronization, whereas individual SPR measures did predict synchronization, consistent with previous findings with musicians^9^. Thus, individuals’ movement rates alone (in both SMT and SPR tasks) did not account for synchronization behavior. One explanation is that SMT provides less sensory feedback for error correction than SPR; timing in SPR tasks has shown evidence of auditory feedback-related error correction^31^. This explanation is consistent with current and previous findings that musically trained individuals exhibit less temporal variability than untrained individuals in rhythmic tasks^47^. SMT and SPR measures may be driven by different constraints; SMT measures are modulated by developmental changes^18^, whereas SPR measures may be modulated more by enhanced auditory exposure gained by musical training. The role of musical training in SMT has been documented with inconclusive or negative outcomes^48,49^, and until recently, SPR was only measured in musically trained individuals. The potential influence of different mechanisms in SMT and SPR is consistent with an interpretation that they reflect different intrinsic movement frequencies, one motoric in origin and the other related to auditory-motor coupling, a hypothesis for future investigation.

The study’s reliance on a one-finger tapping task that both musicians and non-musicians could perform is one limitation of the current study; extensions to more complex movement sequences are necessary. Another limitation is the inclusion of simple familiar melodies, which may not reveal the range of potential influence of sequence structure on the synchronization tasks^32^. For example, the distance between diagonal lines typical of high determinism or predictability (Fig. 6 top) may be influenced by the musical structure. Also important, the sampling of musically trained and untrained individuals does not address the full scale of auditory expertise that individuals obtain. This limitation prevents us from addressing the shift in temporal flexibility that is assumed to occur between the endpoints of the expertise scale, as individuals gain motor skills such as producing a melodic sequence with temporal regularity. Future designs may include a broader range of participants and melodies to track the development of synchronization mechanisms, as well as other novel measurements of rhythmic movement that can be applied to individuals with and without specialized training^50^.

In sum, partners’ interpersonal synchrony in an auditory-motor task is related to each partner’s endogenous rhythms in individual sound production. Consistent with dynamical systems explanations of increased coupling between oscillators with similar frequencies, the timing of intertap intervals as well as asynchronies produced by musically untrained individuals demonstrated more rigidity and predictability than those of musically trained individuals. Both groups of individuals demonstrated constraints of their spontaneous production frequencies in the joint synchronization task, as exhibited by the successful prediction of joint synchronization from the recurrence measures of the partners’ solo performances. The increased temporal variability in non-musicians’ auditory-motor tapping is not simply noise but instead reflects rhythmic patterns that may be overcome with training, as motor and auditory systems become coupled. These novel findings point to two mechanisms driving musicians’ flexibility in timing tasks: first, a honed ability to use auditory feedback in temporal adaptation; and second, an ability to de-couple one’s movement timing from the intrinsic frequencies at which that movement is easiest to produce. These mechanisms can be addressed in future studies that compare performance on a wider variety of auditory rhythms.

## Method

### Participants

Twenty-eight musically trained and 28 untrained adults (18–35 years old) participated. Sample size was estimated from Zamm et al.^8^ findings which used a similar design (2 groups of 20 individuals each with repeated measures, treating pair as random variable) that yielded moderate to large effect sizes (partial η^2^ = 0.25). Inclusion criteria were > 6 years of individual instruction on a musical instrument for musicians (*M* = 10.43 years), and <  = 2 years of instruction for nonmusicians (*M* = 0.42 years). Participants were randomly paired within group (musician/non-musician). All participants exhibited normal hearing (< 30 dB HL) for the stimulus frequency range (125–750 Hz), determined by a pure-tone audiometric screening, and had familiarity with the experimental melodies, evaluated by accurate humming of the melodies. Participants provided written consent and the study was conducted in accordance with the Declaration of Helsinki, the Canadian Tri-Council Policy Statement on Ethical Conduct for Research Involving Humans (TCPS2-2018). The study’s experimental protocols were approved by the local Research Ethics Board of McGill University (REB #1951018). Informed consent was obtained from each participant.

### Materials and equipment

Two familiar melodies were used: *Happy Birthday* (in D Major) and *Twinkle, Twinkle Little Star* (in G Major, referred to as “Twinkle”). Happy Birthday served as a practice melody and Twinkle served in both practice and experimental trials, chosen for its primarily isochronous rhythmic structure. The two partners heard the melody in a different pitch range (one octave apart) for the entire study (for example, Partner A’s melody started on pitch G3 and Partner B’s on pitch G4) to differentiate their parts when they performed together. The melody tones were sounded with a marimba timbre (Roland Studio Canvas GM2 sound bank; #030) and metronome tones with a woodblock percussion timbre (sound bank #206). A sine tone (sound bank #158) signaled the start of trials.

Participants tapped the melody on a force-sensitive resistor connected to an Arduino Uno device that sent MIDI timing signals to the computer running FTAP 2.1.07a^51^. Tones were generated by a Mobile Studio Canvas SD-50 and were heard over studio headphones at a comfortable listening level. Identical equipment was used for the two partners in all conditions. The two Arduino tapping devices were assigned to different MIDI channels and were recorded to FTAP on the same computer (synchronized) during the joint performances (see Supplemental Materials).

### Tasks

The study used a mixed design with two tasks (Solo and Duet) as within-subject factor and musical training as between-subject factor. Solo tasks included a spontaneous motor tempo (SMT) and a spontaneous production rate (SPR) task. During the SMT task, participants tapped at a comfortable, steady rate in the absence of any auditory feedback. During the SPR task, participants tapped the experimental melody at a comfortable, steady rate with auditory feedback. Performances from the SPR task were used to calculate each participant’s mean intertap interval for use as a cued rate in the Duet tasks. Solo tasks always occurred in the order SMT then SPR, to avoid auditory imagery carryover, and Solo tasks preceded Duet tasks. The Duet task was a synchronization-continuation task with two Cued Rate conditions. The initial metronome cue was set to Partner A’s Solo SPR rate in one condition and to Partner B’s Solo SPR in another condition; the order of cued rates was randomly determined. Members of each pair took turns with PartnerA/PartnerB roles.

### Procedure

Each partner completed the Solo tasks in separate testing rooms. First, participants completed the familiar melody assessment and an audiometry screening. Participants then completed the SMT task while seated at a table with the tapping pad (force sensing resistor). They were instructed to use the index finger of their dominant hand to tap at a comfortable, steady rate on the pad. One practice trial and three experimental trials were recorded.

Participants then performed the Solo SPR task with the practice melody (Happy Birthday). They were instructed to tap the melody at a comfortable steady rate, and that each time they produced a tap, the next tone of the melody would sound. Next, they performed the same task with the experimental melody (Twinkle) with the same instruction and were told to repeat the melody several times until they no longer heard auditory feedback, signaling the end of the trial after 3.5 melody repetitions (one repetition = 48 quarter-note beats). They performed a practice trial and three experimental trials. If a participant made gross timing errors (such as starting, restarting, or ending a trial at the wrong time), the trial was discarded and the participant performed another trial (approximately 8% of trials). The first three error-free experimental trials were used (up to six experimental trials were permitted). Participants then completed the musical background questionnaire while each participant’s mean Solo SPR value (mean InterTap Interval, ITI) was computed.

Participants then practiced synchronizing the melody with a metronome cue set to the mean of the two participants’ Solo SPR values; each participant was instructed to wait for 8 metronome beats and then to start synchronizing their taps with the metronome. Then the participants practiced a synchronization-continuation task in which they were instructed to listen to the first 8 metronome beats and to start synchronizing taps with the metronome, which turned off after 8 additional beats. Participants continued tapping the melody at the cued rate until no more auditory feedback was heard (after 3.5 melody repetitions), signaling the end of the trial.

Partners moved to the same testing room for the Duet task where they were seated facing each other over a screen that left the partners’ heads visible but occluded the partners’ bodies below the shoulders. Partners took turns tapping the experimental melody, synchronizing with a metronome set to the average Solo SPR of the pair, so that they became accustomed to the pitch range of each partner (one octave away from their part). Similar to Solo trials, Duet trials began with 8 metronome beats, followed by 8 metronome beats with which they synchronized their taps, and then the metronome ended during the continuation phase while participants continued to tap the melody at the cued rate until the auditory feedback ended (after 3.5 melody repetitions). Partners then completed a practice synchronization trial while the metronome cue was set to PartnerA’s Solo SPR rate, and then a practice synchronization-continuation trial at the same rate. Partners then performed 3 experimental synchronization-continuation trials at PartnerA’s rate, each lasting 3.5 repetitions. If a trial contained timing errors, the trial was ended and a new trial was begun. After three experimental trials were recorded at PartnerA’s Solo SPR, the Duet procedure was repeated with the metronome cue set to Partner B’s Solo SPR until 1 practice trial and 3 experimental synchronization-continuation trials were obtained in each condition (total of 6 experimental trials per pair). The experiment lasted about one hour and participants received a small remuneration.

### Analysis

The mean Spontaneous Motor Tempo (SMT) was calculated as the mean inter-tap-interval (ITI) of the first 30 taps, averaged across trials^18^. The mean Spontaneous Production Rate (SPR) was calculated as the mean ITI of the middle 2 repetitions of each trial, to capture trial positions of maximal stability in performance timing^38,52^, then averaged across trials. Half note ITIs were interpolated, resulting in 96 ITIs per Solo SPR trial. Outlier ITIs more than 3 standard deviations from the mean ITI across trials were discarded (less than 1% of total ITIs in SMT; 0.007% of musicians’ total ITIs and 1.44% of non-musicians’ ITIs). The coefficient of variation (CV) was calculated for SMT and SPR trials as the standard deviation of ITIs divided by the mean ITI for 30 taps (taps 17–46 of the SPR task).

Synchronization in the Duet task was measured by the tap onset time of the partner whose SPR rate was cued minus the other partner’s tap onset time. A negative asynchrony means that the partner whose SPR served as the cued rate tapped earlier than their partner. Asynchrony outliers 3 standard deviations or more from the mean asynchrony were discarded (1.54% of asynchronies in musicians and 1.57% in non-musicians). Solo and Duet measures (ITIs and asynchronies) were analyzed with analyses of variance (ANOVA) tests of group differences (musician/non-musician) and task differences (SPR/SMT). Pearson correlation coefficients were computed to analyze comparisons between behavioral (ITI, asynchrony) and RQA measures (recurrence, determinism) in Solo and Duet tasks.

Recurrence Quantification Analyses (RQA) were performed on Solo (ITI) and Duet (asynchrony) measures. RQA is a non-linear time series analysis that uncovers recurring patterns and is especially useful for non-stationary measures^31,53^. RQA was applied to data from entire trials (without outlier removal) to maximize the times series length. RQA analyses of Solo performances included 126 ITIs (excluding the first 16 ITIs that included metronome beats and the last 2 ITIs) and Duet performances included 111 asynchronies (the same range as the Solo performances, excluding the first 14 asynchronies that included metronome beats and the last asynchrony). RQA metrics of recurrence rate (probability that a specific state will recur in the time series, indicated by points in the recurrence plot) and determinism (predictability of the behavioral pattern, captured by the proportion of recurrent points that form a continuous diagonal line) were analyzed. The RQA minimum length parameter was set to = 2 to incorporate all pattern lengths. The delay parameter, estimated from the first minimum in mutual information function, was set to = 2. The number of embedding dimensions, which was determined by values necessary for the false-nearest neighbor function to approach zero^54^, was set to 4 for Solo analyses and to 3 for Duet analyses. The radius was fixed to = 1 which yielded recurrence rates averaging around 10%, consistent with recommended values for behavioral data^54^. Recurrence quantification analyses were based on the CRP Toolbox^55,56^. The nonlinear RQA metrics were analyzed with nonparametric tests of group differences (Mann–Whitney U).

### Ethical approval

Before the experiment, participants were provided with an information sheet that outlined the general purpose of the study and informed them that they could withdraw at any time without penalty. All methods were reviewed by the Ethics Research Board of McGill University and were in accordance with the Declaration of Helsinki.

## Supplementary Information

Supplementary Information.

## Data Availability

The datasets generated and analysed during the current study are available from the corresponding author in an anonymized format upon reasonable request.
