# Supplementary material for: Endogenous rhythms influence musicians’ and non-musicians’ interpersonal synchrony
Source: Sci Rep. 2022 Jul 28;12:12973. doi: 10.1038/s41598-022-16686-2 (PMC9334298; doi:10.1038/s41598-022-16686-2)
Supplement: Supplementary file 1 — Supplementary Information. [file 41598_2022_16686_MOESM1_ESM.docx]

Endogenous Rhythms Influence

Musicians’ and Non-musicians’ Interpersonal Synchrony:

Supplemental Materials

Pauline Tranchant, Eléonore Scholler & Caroline Palmer

McGill University

**Equipment:**

The temporal response characteristics of the force sensor-to-Arduino devices and Arduino signals-to-FTAP software were measured for the two tapping pads (#1 and #2) with a Tektronix TDS 2002 oscilloscope. The time elapsed from when the tap on the force sensitive resistor (FSR) reaches threshold (0.73V) to the start of the MIDI signal sent from the Arduino was first measured (see Supplementary Figure 1). Data from 100 taps on each pad confirmed that this duration was on average 571.9 µs (SD = 35 µs) for tapping pad #1 and 591.8 µs (SD = 30 µs) for tapping pad #2 (see Supplementary Figure 1).

The time elapsed from the start of the MIDI signal sent out from the Arduino to the start of the MIDI signal sent out from FTAP via an M-Audio UNO MIDI-USB connector was also measured. Data from 100 taps on each pad confirmed that this duration was on average 1.48 ms (SD = 0.15 ms) for tapping pad #1 and 1.38 ms (SD = .14 ms) for tapping pad #2 (see Supplementary Figure 2).


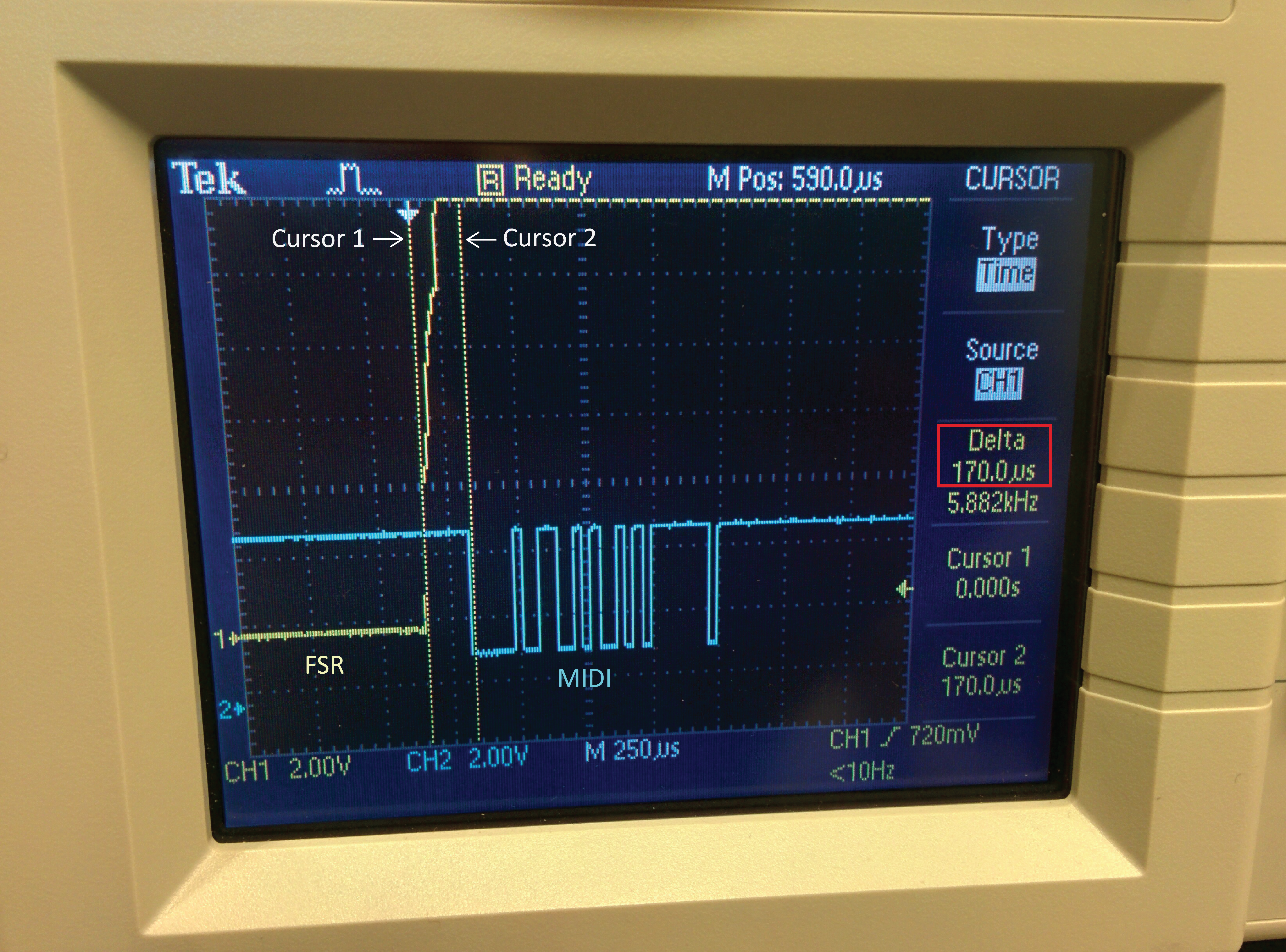


*Supplementary Figure 1.* One sample trial measuring the time from when the tap reaches threshold on the FSR (yellow trace) to the start of the MIDI signal sent out from the Arduino (blue trace). Cursor 1 is aligned with the time at which the tap reaches threshold on the FSR, and cursor 2 is aligned with the time at which the MIDI signal begins. The Delta value (170 µs, marked by the red box) displays the difference between cursors 1 and 2.


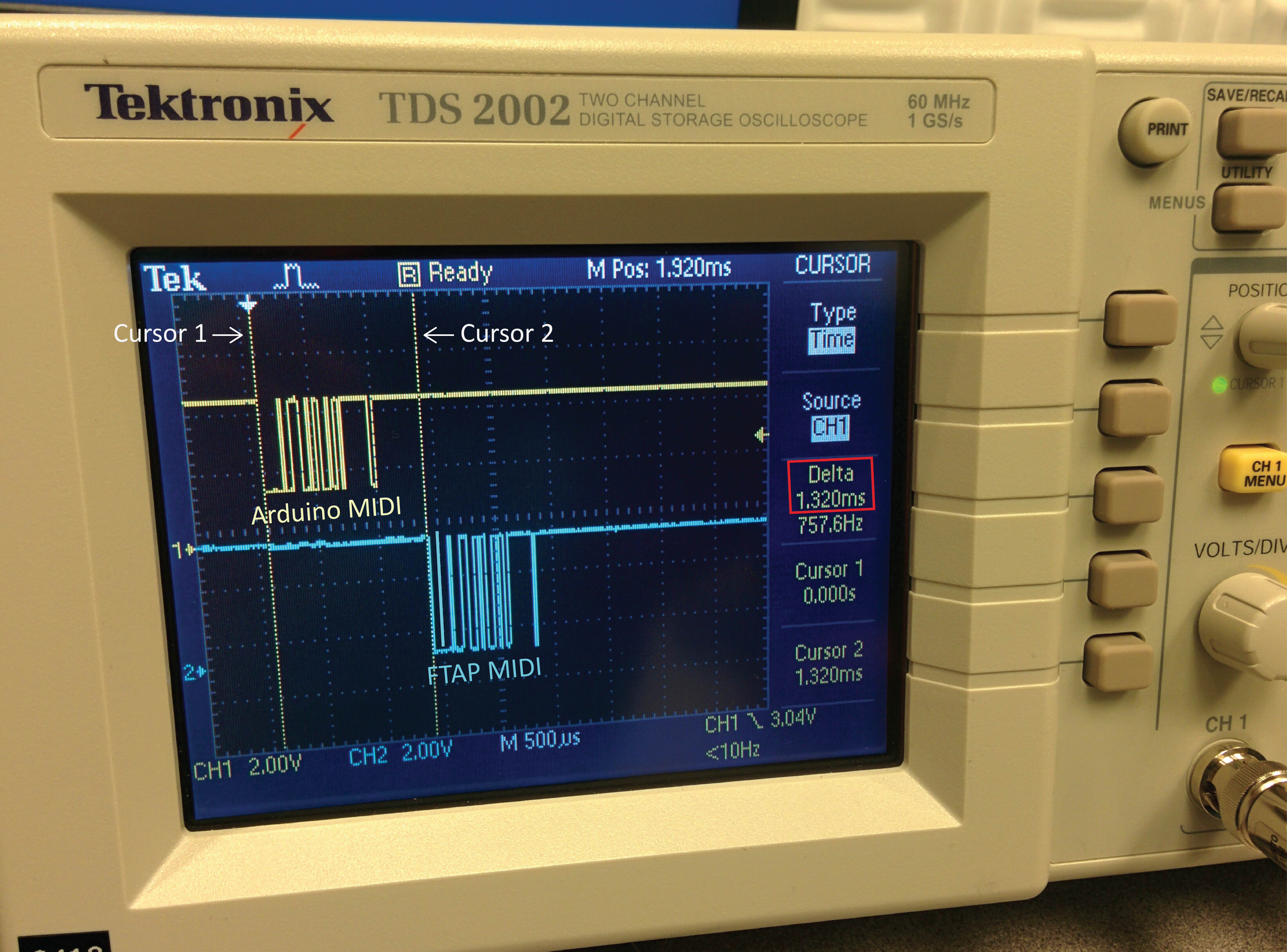


*Supplementary Figure 2.* One sample trial measuring the time from the start of the MIDI signal sent out from the Arduino (yellow trace) to the start of the MIDI signal sent out from FTAP (blue trace). Cursor 1 is aligned with the time of the start of the MIDI signal sent out from the Arduino, and cursor 2 is aligned with the time of the start of the MIDI signal sent out from FTAP. The Delta value (1.32 ms, marked by the red box) calculates the elapsed time between cursors 1 and 2.
